# Short-Term Clinical Results of Minimally Invasive Direct Coronary Artery Bypass (MIDCAB) Procedure

**DOI:** 10.3390/jcm13113124

**Published:** 2024-05-26

**Authors:** Eissa Alaj, Vahid Seidiramool, Veaceslav Ciobanu, Farhad Bakhtiary, Nadejda Monsefi

**Affiliations:** Department of Cardiac Surgery, University Hospital Bonn, 53127 Bonn, Germanyvahid.seidiramool@ukbonn.de (V.S.);

**Keywords:** minimally invasive cardiac surgery, off-pump CABG, endoscopic harvesting of mammary artery

## Abstract

**Objectives:** Minimally invasive direct coronary artery bypass (MIDCAB) is an alternative for revascularisation of the isolated left anterior descending (LAD) artery or as a multi-vessel (MV) procedure for the diagonal branch (RD) or the left circumflex coronary artery (LCX) region. **Methods:** From 2021 to 2022, 91 patients underwent MIDCAB or multi-vessel MIDCAB procedures in our heart center. The left internal mammary artery (LIMA) was anastomosed to the left anterior descending artery via the left minithoracotomy approach in all patients. **Results:** Of the patients, a total of 86.8% were male. Eighty percent of the patients had two- or three-vessel coronary artery disease. The mean age was 65.1 ± 10.1 years. The mean operation time was 2.6 ± 0.8 h. The 30-day mortality was 0. The mean required packed red blood cells (pRBC) was 0.4 ± 1.2 unit. The mean intensive care unit stay (ICU) was 1.5 ± 1.6 days. The mean follow-up time was 1.5 ± 0.5 years. One patient received percutaneous coronary intervention due to de novo stenosis of the RCA. Late mortality was 2.2%. The Kaplan–Meier survival rate was 98.8% at 1 and 2 years. **Conclusions:** The postoperative complication rate of our MIDCAB cohort is low, and the short-term survival is favorable. Our postoperative and short-term clinical results demonstrate that this procedure is safe and feasible.

## 1. Introduction

Coronary artery disease (CAD) remains one of the leading causes of morbidity and mortality worldwide [1]. The primary treatment approach for CAD has been revascularization, aiming to restore the blood flow to the ischemic myocardium. Historically, coronary artery bypass grafting (CABG) via median sternotomy has been the gold standard for multi-vessel disease, offering favorable, long-term outcomes [2]. However, with advances in technology and surgical techniques, minimally invasive approaches have emerged as promising alternatives, seeking to reduce surgical trauma and enhance postoperative recovery [3].

Minimally invasive direct coronary artery bypass (MIDCAB) is a procedure that provides direct access to the heart through a small thoracotomy, eliminating the need for sternotomy [4]. It has been particularly favored for the revascularization of the left anterior descending artery (LAD). The MIDCAB procedure is also an option as a hybrid coronary revascularization (HCR) in the setting of an incomplete surgical revascularization for selected patients. Furthermore, MIDCAB surgery can be applied as a multi-vessel procedure for several coronary territories like the RD or LCX [5]. The benefits of the MIDCAB technique have been reported, which include less surgical trauma, reduced operative bleeding and fast recovery [6,7,8]. However, it remains technically challenging and demands meticulous patient selection and an experienced surgical team for optimal outcomes. This study aims to analyze the short-term clinical results of our single center experience with the MIDCAB procedure and to evaluate the safety and feasibility of this technique.

## 2. Material and Methods

### 2.1. Study Design and Population

In this study, we analyzed the clinical results of 91 patients who underwent a MIDCAB procedure in the Department of Cardiac Surgery at the University Hospital Bonn from January 2021 to December 2022, retrospectively. Patients were clinically followed up via telephone. Follow-up data, including coronary angiography results, were retrieved from referring cardiologists. This study was approved by the local ethics committee (#446/21).

### 2.2. Patient Selection Criteria 

The inclusion criteria for the MIDCAB procedure were relevant stenosis or occlusion of the proximal or medial LAD. In patients with two or three vessel CAD, the RD, LCX, or ramus intermedius (RIM) were targets for multi-vessel (MV) MIDCAB revascularization. In patients who underwent HCR, the right coronary artery and/or RCX were treated with PCI, postoperatively. The following circumstances were exclusion criteria for the MIDCAB procedure: chest radiation, left thoracotomy (for lung or breast surgery), redo CABG in patients’ medical history, stenosis of the left subclavian artery, emergency operation, and/or hemodynamically unstable patients.

### 2.3. Surgical Technique

**Figure 1:**
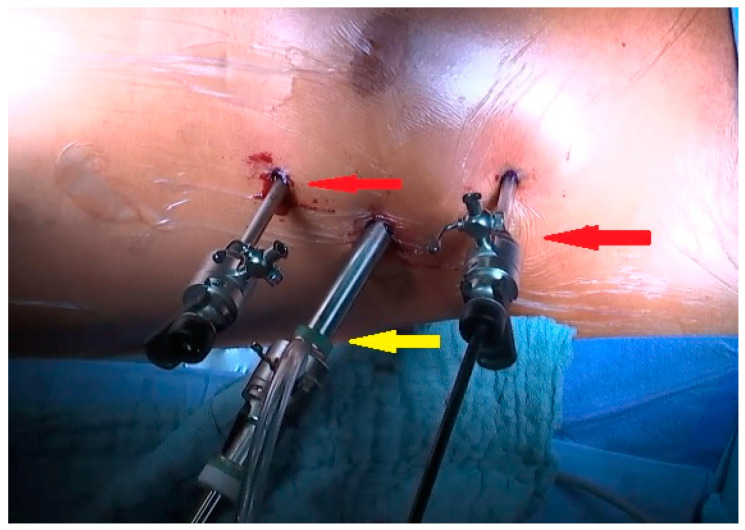
Port access for endoscopic harvesting of the left internal mammary artery. Red arrows: Port access for hook electrocautery and forceps. Yellow arrow: Port for 3D endoscope.

**Figure 2:**
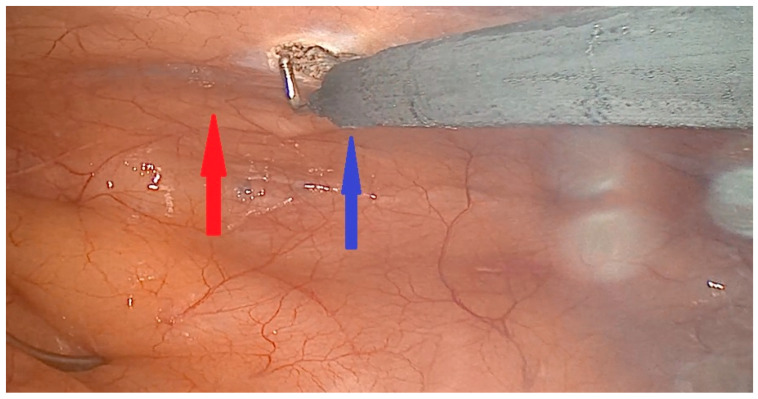
Endoscopic harvesting of the left internal mammary artery. Red arrow: Left internal mammary artery. Blue arrow: Hook electrocautery.

**Figure 3:**
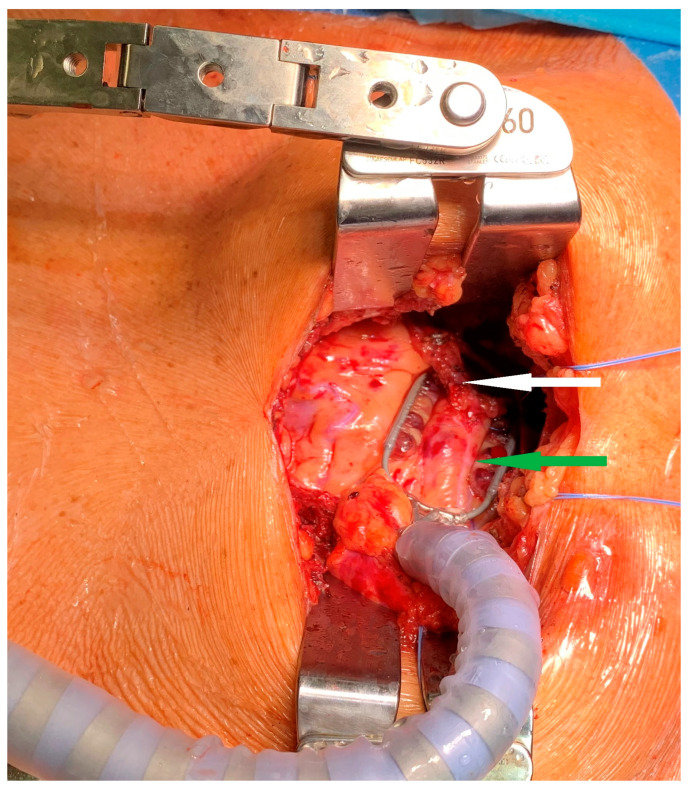
MIDCAB: Intraoperative view showing LIMA (white arrow) to LAD (green arrow) anastomosis.

We already published the surgical technique for a MIDCAB procedure [9]. In short, the patients were placed in a supine position, with a 30° elevation of the left thorax. They were intubated with a double-way endotracheal tube to deflate the left lung. In case of endoscopic harvesting of the left internal mammary artery (LIMA), 3 incisions for port access were performed (Figure 1 and Figure 2) to place the 3D endoscope, the forceps, and the hook electrocautery. For open harvesting of the LIMA, a small skin incision was performed, and the left pleural space was opened through the 4th or 5th intercostal space. With the help of a MICS retractor for LIMA (lifting retractor, Geister, Tuttlingen, Germany), a pedicled LIMA graft was harvested. Then, a systemic heparinization was given, the pericardium was opened, and the LAD was localized. The distal anastomosis was performed in an off-pump technique with the help of a vacuum tissue stabilizer (Octopus Evolution, Medtronic, Minneapolis, MN, USA), Figure 3, and an intracoronary shunt (Medtronic, Minneapolis, MN, USA). Intraoperative bypass flow was measured routinely. In MV-MIDCAB, a segment of saphenous vein was harvested from the lower leg. The venous graft was anastomosed to the target vessel in the same way. A proximal anastomosis (saphenous vein graft as T-Graft to LIMA) was performed. Subsequently, protamin was given 1:1, a thorax drainage was inserted into the left pleura, and the thoracotomy was closed. Three consultant cardiac surgeons performed the MIDCAB procedures in our department. 

### 2.4. Postoperative Care and Clinical Follow-Up

Hemodynamic parameters, wound healing, and potential postoperative complications like infections or bleeding were documented. A systematic clinical follow-up regarding the health status and postoperative complications was established via phone call. Follow-up data including coronary angiography results were retrieved from referred cardiologists. 

### 2.5. Statistical Analysis

Statistical analyses were calculated with the biometrically analysis of sampling software (BIAS 11.06, Frankfurt, Germany). Categorical data were presented as percentages, and continuous data were described as mean value ± standard deviation. Survival was analyzed with the Kaplan–Meier method.

## 3. Results

### 3.1. Preoperative Data

The patients´ preoperative characteristics are presented in Table 1. The majority of the patients (53.8%) presented with three-vessel CAD. 

### 3.2. Operative and Postoperative Results

The operative and postoperative results are listed in Table 2. MV-MIDCAB was performed in 14 patients (15.4%). Four patients underwent rethoracotomy because of bleeding (thoracic muscle). One patient underwent re-coronary angiography due to myocardial infarction postoperatively. The LIMA-LAD anastomosis was unremarkable. This patient received PCI with a stent in the diagonal branch (relevant stenosis).

### 3.3. Follow-Up Results

**Figure 4:**
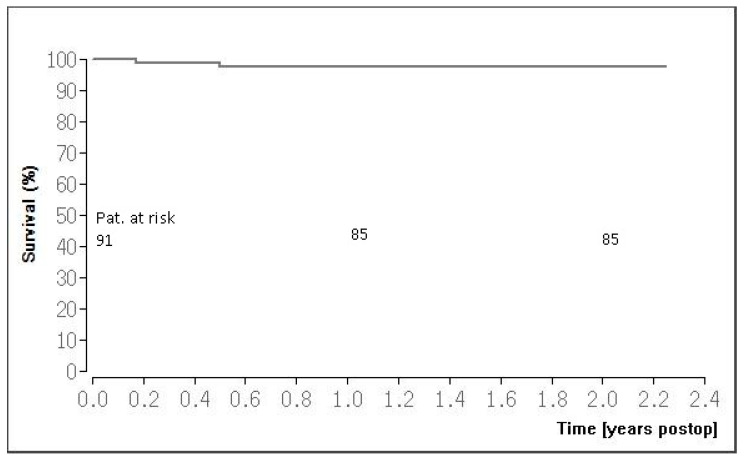
Kaplan–Meier survival curve.

The follow-up results are listed in Table 3. The mean follow-up time was 1.5 ± 0.5 years. Follow-up was completed in 89 patients (97.8%). One patient underwent re-coronary angiography due to de novo stenosis of the RCA. The Kaplan–Meier survival rate was 98.8% at 1 and 2 years (Figure 4). 

## 4. Discussion

The paradigm of coronary revascularization is continuously evolving, with minimally invasive direct coronary artery bypass (MIDCAB) surgery emerging as a promising alternative to traditional CABG, for selected patients [10]. Our study, predominantly involving a male population (87%), aligns with the broader epidemiological trend of higher coronary artery disease prevalence in men [11].

The 30-day mortality rate is 0% in our cohort, and the postoperative complication rates regarding bleeding (4.4%), neurologic event (0%), and wound infection (2.2%) are low. These findings underscore the safety and favorable in-hospital clinical outcomes associated with the MIDCAB procedure.

Similar results regarding the mortality rate were published before: Raja and colleagues reported a 30-day mortality rate of 2% among 668 patients undergoing the MIDCAB procedure [12], while Holzhey noted a mortality of 0.8% in 1768 patients [13]. Calafiore et al. observed a 30-day mortality of 0.6% in 155 patients who underwent a MIDCAB procedure [14]. We observed no cases of neurological events in our cohort, which is an excellent result. Manuel and colleagues reported a neurological rate of 1.8% in their MIDCAB [15]. The rethoracotomy (for bleeding) rate in our study is 4.4%, that is comparable to published data which ranges between 1 and 4% [15,16]. Wound healing disturbances were observed in only 2.2% of our cohort, which is comparable to other studies, including 3.4% reported by Reuthebuch and colleagues in patients undergoing MIDCAB procedures [16] and 2.4% reported by Raja [12].

The average ICU stay in our study is 1.5 ± 1.6 days, which is similar to the results reported by Reuthebuch [16]. We observed a ventilation time of 4.0 ± 6.0 h, which is short. There are published data regarding the ventilation time of MIDCAB patients, which is about 24 h [16].

However, it is important to note that in Manuel’s study, 1.8% of patients were converted to sternotomy [15], while our study had a 0% conversion rate. These findings underline the feasibility and procedural safety of the MIDCAB procedure. However, MIDCAB is technically demanding. Therefore, optimal patient selection and the expertise of the surgical team are crucial.

In our study, we observed one patient (1.1%) who required reintervention due to de novo stenosis of the RCA during the follow-up period. This is comparable to the reintervention rate for anastomosis stenosis of 1% reported by Reuthebuch [16] and 0.7% reported by Manuel [15]. Holzhey and colleagues reported a reintervention rate of 3.3% [13]. Our short-term survival rate of 98.8% at 1 and 2 years postoperatively is an excellent outcome. These follow-up results are comparable with published data ranging from 92% to 96% at mid-term survival [15,16]. At the latest follow-up, the majority of our patients (95.5%) were classified in the CCS class of 0, emphasizing the favorable clinical outcomes of our cohort. There are some limitations regarding our study. We present the results at a single center, and the number of our patient cohort is small (n = 91). There were 2 patients lost to follow-up that could have an impact on the outcome.

## 5. Conclusions

The postoperative complication rate of our MIDCAB cohort is low. We demonstrate a 30-day mortality rate and a conversion rate of 0. The observed survival rates of 98.8% at 1 and 2 years postoperatively are outstanding in this study. Our postoperative and short-term clinical results confirm that the MIDCAB procedure is safe and feasible.

## Figures and Tables

**Table 1:** Preoperative patient characteristics.

Parameter	
Number of patients, n	91
Age, M ± SD (years)	65.1 ± 10.1
Gender, male n (%)	79 (86.8%)
NYHA class 1	0
NYHA class 2	16 (17.6%)
NYHA class 3	71 (78.0%)
NYHA class 4	4 (4.4%)
CCS class 0	0
CCS class 1	3 (3.3%)
CCS class 2	23 (25.3%)
CCS class 3	62 (68.1%)
CCS class 4	3 (3.3%)
CAD 1 Vessel, n (%) CAD 2 Vessels, n (%) CAD 3 Vessels, n (%)	18 (19.7%) 24 (26.3%) 49 (53.8%)
Ejection Fraction, M ± SD (%) Comorbidities	50.2 ± 10.7
Diabetes mellitus, n (%)	26 (28.6%)
COPD, n (%)	9 (9.9%)
Renal insufficiency, n (%)	8 (8.8%)
Myocardial infarction, n (%)	29 (31.9%)
Arterial hypertension, n (%)	63 (69.2%)
EUROScore I log., (M ± SD %)	5.2 ± 11.2
Peripheral Arterial Disease (PAD), n (%)	19 (20.9%)
Stent, n (%)	14 (15.4%)

M: mean; SD: standard deviation; COPD: chronic obstructive pulmonary disease; CAD: coronary artery disease; NYHA: New York Heart Association; CCS: Canadian Cardiovascular Society classification.

**Table 2:** Operative and perioperative data.

Operative	
One coronary art anastomosis, n (%)	77(84.6%)
Two coronary artery anastomoses, n (%)	10 (11.0%)
Three coronary artery anastomoses, n (%)	4 (4.4%)
Operation time, M ± SD (h)	2.6 ± 0.8
Conversion to sternotomy, n (%)	0
Number of anastomoses, n	109
postoperative	
Intensive care unit (ICU) length of stay M ± SD (Days)	1.5 ± 1.6
Ventilation time, M ± SD (hours)	4.0 ± 6.0
Rethoracotomy, n (%)	4 (4.4%)
Stroke, n (%)	0
Wound infection, n (%)	2 (2.2%)
30-day mortality, n (%)	0
Myocardial infarction, n (%)	1 (1.0%)
Stent, n (%)	1 (1.0%)
Coronary angiography, n (%)	1 (1.0%)
Blood transfusion (pRBCs), n (%)	0.4 ± 1.2
Complete revascularization, n (%)	73 (80.2%)
Hybrid revascularization, n (%)	18 (19.8%)

Mean; SD: standard deviation; pRBC: packed red blood cells.

**Table 3:** Follow-up data.

Parameter	
Number of patients, n	89
NYHA class 1	78 (87.6%)
NYHA class 2	11 (12.4%)
NYHA class 3	0
NYHA class 4	0
CCS class 0	85 (95.5%)
CCS class 1	4 (4.5%)
CCS class 2	0
CCS class 3	0
CCS class 4	0
Coronary angiography, n (%) Stent, PCI n (%) Mortality, n (%)	1 (1.1%) 1 (1.1%) 2 (2.2%)
Myocardial infarction, n (%)	0

NYHA: New York Heart Association; PCI: percutaneous coronary intervention.

## Data Availability

Data is unavailable due to privacy or ethical restrictions.
